# Crystal structure of (4*E*)-4-(8-meth­oxy-2*H*-chromen-2-yl­idene)-3-methyl-1-phenyl-1*H*-pyrazol-5(4*H*)-one

**DOI:** 10.1107/S2056989015009445

**Published:** 2015-05-23

**Authors:** Muhammad Salim, Munawar Ali Munawar, Muhammad Nawaz Tahir, Muhammad Shahid, Khizar Iqbal Malik

**Affiliations:** a Department of Chemistry, University of the Punjab, Lahore, Punjab, Pakistan; b Department of Physics, University of Sargodha, Sargodha, Punjab, Pakistan

**Keywords:** crystal structure, pyrazolone, π–π inter­actions

## Abstract

In the title compound, C_20_H_16_N_2_O_3_, the phenyl substituent attached to the pyrazole ring makes a dihedral angle of 4.87 (7)° with the rest of the mol­ecule. In the crystal, mol­ecules are connected into inversion dimers of the *R*_2_^2^(14) type by pairs of C—H⋯O inter­actions. π–π inter­actions exist between the benzene and pyrazole rings at a distance of 3.701 (1) Å. Similarly, π–π inter­actions are present at a centroid–centroid distance of 3.601 (1) Å between the oxygen-containing heterocyclic ring and meth­oxy substituted aromatic ring of a neighbouring mol­ecule. Additional C—H⋯π and C=O⋯π inter­actions are also observed.

## Related literature   

For related structures, see: Chaudhry *et al.* (2012 ▸); Holzer *et al.* (1999 ▸); Malik *et al.* (2009 ▸).
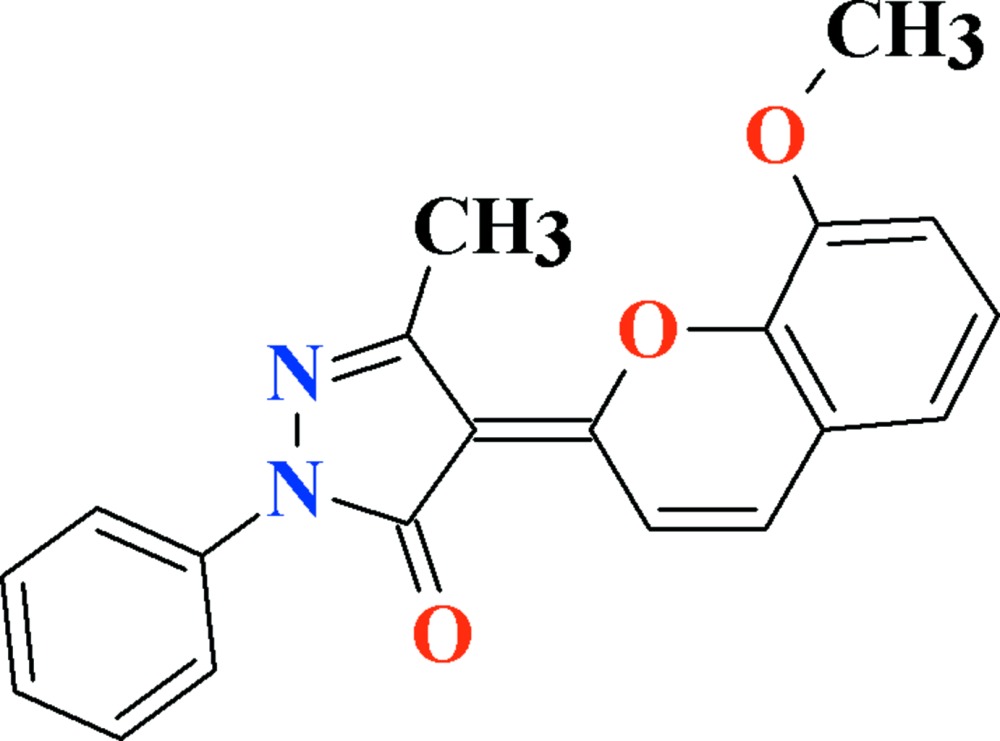


## Experimental   

### Crystal data  

C_20_H_16_N_2_O_3_
*M* *_r_* = 332.35
Monoclinic, 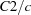

*a* = 28.179 (5) Å
*b* = 4.7108 (8) Å
*c* = 23.819 (5) Å
β = 92.957 (7)°
*V* = 3157.7 (10) Å^3^
*Z* = 8
Mo *K*α radiation
μ = 0.10 mm^−1^
*T* = 296 K
0.40 × 0.22 × 0.18 mm

### Data collection  

Bruker Kappa APEXII CCD diffractometer
Absorption correction: multi-scan (*SADABS*; Bruker, 2005 ▸) *T*_min_ = 0.961, *T*_max_ = 0.985
13056 measured reflections
3419 independent reflections
2389 reflections with *I* > 2σ(*I*)
*R*_int_ = 0.033

### Refinement  

*R*[*F* ^2^ > 2σ(*F* ^2^)] = 0.042
*wR*(*F* ^2^) = 0.127
*S* = 1.06
3419 reflections
229 parameters
H-atom parameters constrained
Δρ_max_ = 0.22 e Å^−3^
Δρ_min_ = −0.16 e Å^−3^

### 

Data collection: *APEX2* (Bruker, 2007 ▸); cell refinement: *SAINT* (Bruker, 2007 ▸); data reduction: *SAINT*; program(s) used to solve structure: *SHELXS97* (Sheldrick, 2008 ▸); program(s) used to refine structure: *SHELXL2014* (Sheldrick, 2015 ▸); molecular graphics: *ORTEP-3 for Windows* (Farrugia, 2012 ▸) and *PLATON* (Spek, 2009 ▸); software used to prepare material for publication: *WinGX* (Farrugia, 2012 ▸) and *PLATON*.

## Supplementary Material

Crystal structure: contains datablock(s) global, I. DOI: 10.1107/S2056989015009445/im2465sup1.cif

Structure factors: contains datablock(s) I. DOI: 10.1107/S2056989015009445/im2465Isup2.hkl

Supporting information file. DOI: 10.1107/S2056989015009445/im2465Isup3.cml

**Fig. 1.**
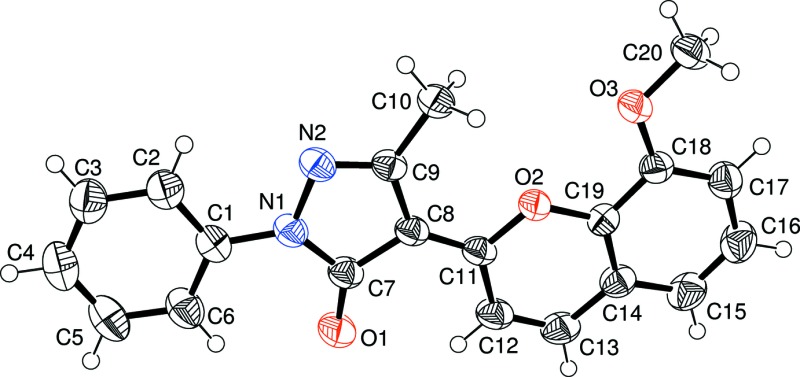
View of the title compound with the atom numbering scheme. Thermal ellipsoids are drawn at the 50% probability level. H-atoms are shown by small circles of arbitrary radii.

. DOI: 10.1107/S2056989015009445/im2465fig1.tif
View of the title compound with the atom numbering scheme. Thermal ellipsoids are drawn at the 50% probability level. H-atoms are shown by small circles of arbitrary radii.

**Fig. 2.**
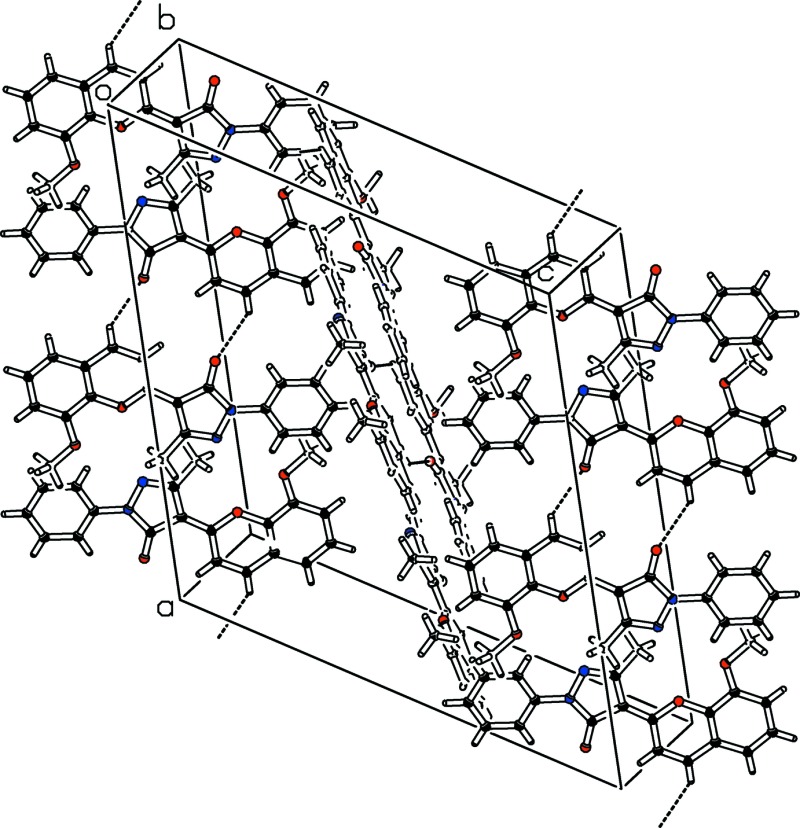
Partial packing (PLATON; Spek, 2009) which shows that molecules are dimerized due to C—H···O bondings.

PLATON . DOI: 10.1107/S2056989015009445/im2465fig2.tif
Partial packing (*PLATON*; Spek, 2009) which shows that mol­ecules are dimerized due to C—H⋯O bondings.

CCDC reference: 1401584

Additional supporting information:  crystallographic information; 3D view; checkCIF report

## Figures and Tables

**Table 1:** Hydrogen-bond geometry and CH and CO interactions (, ) *Cg*1 and *Cg*2 are the centroids of the N1/N2/C7C9 and C11C14/C19/O2 rings, respectively.

*D*H*A*	*D*H	H*A*	*D* *A*	*D*H*A*
C6H6O1	0.93	2.28	2.911(2)	124
C12H12O1	0.93	2.38	3.004(2)	124
C13H13O1^i^	0.93	2.53	3.2577(19)	136
C10H10*A* *Cg*1^ii^	0.96	2.79	3.6812(17)	155
C7O1*Cg*2^iii^	1.23(1)	3.65(1)	3.9797(18)	96(1)

Symmetry codes: (i) 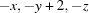; (ii) 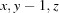; (iii) 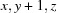.

## supplementary crystallographic information

### S1. Comment

The crystal structures of 5-methyl-2-phenyl-4-((*E*)-3-phenyl-2-hydroxy- prop-2-enylidene)-1,2-dihydro-3*H*-pyrazol-3-one (Holzer *et al.*, 1999), (4*Z*)-4-((2*E*)-1-hydroxy-3-(4-methoxyphenyl)prop-2-en-1- ylidene)-3-methyl-1-phenyl-1*H*-pyrazol-5(4*H*)-one (Malik *et al.*, 2009) and (4*Z*)-4-((2*E*)-1-hydroxy-3-(3-nitrophenyl)prop- 2-en-1-ylidene)-3-methyl-1-(4-methylphenyl)-1*H*-pyrazol-5(4*H*)-one (Chaudhry, *et al.*, 2012) have been published which are related to the title compound (I, Fig. 1). No crystal structure has been found containing a 8-methoxy-2*H*-chromene subunit. (I) is synthesized for the biological studies *etc*.

In (I), the benzene ring A (C1–C6) and the (4E)-4-(8-methoxy-2H-chromen-2-ylidene)-5-methyl-2,4-dihydro-3H-pyrazol-3-one (C7 –C20/N1/N2/O1/O2/O3) part of the molecule are planar with r. m. s. deviations of 0.0053 and 0.0108 Å, respectively. The dihedral angle between A/B is 4.87 (7)°. The molecules are dimerized due to C—H···O interactions completing *R*_2_^2^ (14) Molecules are connected to inversion dimers dimerized of the *R*_2_^2^ (14) type by C—H···O interactions. There exist π–π interactions at a distance of 3.7011 (12) Å between the centeroids of *Cg*1—*Cg*3^i^ and *Cg*3—*Cg*1^ii^ [i = *x*, - 1 + *y*, *z* and ii = *x*, 1 + *y*, *z*], where *Cg*1 and *Cg*3 are the centroids of heterocyclic ring *C* (N1/N2/C7/C8/C9) and benzene ring *A*, respectively. Similarly, there exist π–π interaction at a distance of 3.6012 (11) Å between the centeroids of *Cg*2—*Cg*4^ii^ and *Cg*4—*Cg*2^i^ [i = *x*, - 1 + *y*, *z* and ii = *x*, 1 + *y*, *z*], where *Cg*2 and *Cg*4 are the centroids of heterocyclic ring *D* (C11—C14/C19/O2) and methoxy containing benzene ring *E* (C14—C19), respectively. There exist C—H···π and C=O···π interactions (Table 1).

### S2. Experimental

For the preparation of title compound (I), a mixture of 4-acetyl-3-methyl-1-phenyl-5-hydroxy pyrazole (0.218 g, 1 mmoL), 2-methoxybenzaldehyde (0.205 g, 1.5 mmoL) was refluxed for 8 h in glacial acetic acid (15 ml) and concentrated sulfuric acid (0.2 ml). The reaction mixture was diluted with distilled water (60 ml). The precipitate was filtered, washed with methanol and dried. The crude product was purified by column chromatography using n-hexane and ethyl acetate mixtures as eluents. The product was recrystallized using n-hexane to afford red needles (yield = 63%, m.p. 503 K)

### S3. Refinement

H-atoms were positioned geometrically (C–H = 0.93–0.96 Å) and refined as riding with *U*_iso_(H) = *xU*_eq_(C, O), where *x* = 1.5 for methyl and *x* =1.2 for aromatic H-atoms.

### Crystal data

**Table:**

C_20_H_16_N_2_O_3_	*F*(000) = 1392
*M**_r_* = 332.35	*D*_x_ = 1.398 Mg m^−^^3^
Monoclinic, *C*2/*c*	Mo *K*α radiation, λ = 0.71073 Å
*a* = 28.179 (5) Å	Cell parameters from 2389 reflections
*b* = 4.7108 (8) Å	θ = 2.9–27.0°
*c* = 23.819 (5) Å	µ = 0.10 mm^−^^1^
β = 92.957 (7)°	*T* = 296 K
*V* = 3157.7 (10) Å^3^	Needle, red
*Z* = 8	0.40 × 0.22 × 0.18 mm

### Data collection

**Table:**

Bruker Kappa APEXII CCD diffractometer	3419 independent reflections
Radiation source: fine-focus sealed tube	2389 reflections with *I* > 2σ(*I*)
Graphite monochromator	*R*_int_ = 0.033
Detector resolution: 7.70 pixels mm^-1^	θ_max_ = 27.0°, θ_min_ = 2.9°
ω scans	*h* = −35→35
Absorption correction: multi-scan (*SADABS*; Bruker, 2005)	*k* = −6→5
*T*_min_ = 0.961, *T*_max_ = 0.985	*l* = −24→30
13056 measured reflections	

### Refinement

**Table:**

Refinement on *F*^2^	Secondary atom site location: difference Fourier map
Least-squares matrix: full	Hydrogen site location: inferred from neighbouring sites
*R*[*F*^2^ > 2σ(*F*^2^)] = 0.042	H-atom parameters constrained
*wR*(*F*^2^) = 0.127	*w* = 1/[σ^2^(*F*_o_^2^) + (0.0648*P*)^2^ + 0.6123*P*] where *P* = (*F*_o_^2^ + 2*F*_c_^2^)/3
*S* = 1.06	(Δ/σ)_max_ < 0.001
3419 reflections	Δρ_max_ = 0.22 e Å^−^^3^
229 parameters	Δρ_min_ = −0.16 e Å^−^^3^
0 restraints	Extinction correction: *SHELXL2014* (Sheldrick, 2015), Fc^*^=kFc[1+0.001xFc^2^λ^3^/sin(2θ)]^-1/4^
Primary atom site location: structure-invariant direct methods	Extinction coefficient: 0.0021 (4)

### Special details

**Table:**

Geometry. All e.s.d.'s (except the e.s.d. in the dihedral angle between two l.s. planes) are estimated using the full covariance matrix. The cell e.s.d.'s are taken into account individually in the estimation of e.s.d.'s in distances, angles and torsion angles; correlations between e.s.d.'s in cell parameters are only used when they are defined by crystal symmetry. An approximate (isotropic) treatment of cell e.s.d.'s is used for estimating e.s.d.'s involving l.s. planes.
Refinement. Refinement of *F*^2^ against ALL reflections. The weighted *R*-factor *wR* and goodness of fit *S* are based on *F*^2^, conventional *R*-factors *R* are based on *F*, with *F* set to zero for negative *F*^2^. The threshold expression of *F*^2^ > σ(*F*^2^) is used only for calculating *R*-factors(gt) *etc*. and is not relevant to the choice of reflections for refinement. *R*-factors based on *F*^2^ are statistically about twice as large as those based on *F*, and *R*-factors based on ALL data will be even larger.

### Fractional atomic coordinates and isotropic or equivalent isotropic displacement parameters (Å^2^)

**Table:**

	*x*	*y*	*z*	*U*_iso_*/*U*_eq_	
O1	0.06562 (4)	1.0410 (2)	0.06364 (5)	0.0601 (4)	
O2	0.11680 (3)	0.3975 (2)	−0.05229 (4)	0.0405 (3)	
O3	0.16619 (4)	0.0352 (2)	−0.10799 (5)	0.0504 (3)	
N1	0.14466 (4)	0.9876 (2)	0.09568 (5)	0.0407 (3)	
N2	0.18451 (4)	0.8288 (2)	0.08158 (6)	0.0417 (3)	
C1	0.14967 (6)	1.1800 (3)	0.14142 (6)	0.0410 (4)	
C2	0.19424 (6)	1.2193 (3)	0.16813 (7)	0.0501 (4)	
H2	0.2202	1.1180	0.1563	0.060*	
C3	0.19992 (7)	1.4086 (4)	0.21225 (8)	0.0575 (5)	
H3	0.2298	1.4329	0.2301	0.069*	
C4	0.16197 (8)	1.5617 (4)	0.23019 (8)	0.0593 (5)	
H4	0.1661	1.6913	0.2595	0.071*	
C5	0.11808 (7)	1.5206 (4)	0.20427 (8)	0.0619 (5)	
H5	0.0923	1.6222	0.2164	0.074*	
C6	0.11134 (7)	1.3295 (3)	0.16006 (7)	0.0541 (5)	
H6	0.0812	1.3025	0.1431	0.065*	
C7	0.10494 (5)	0.9304 (3)	0.06052 (7)	0.0410 (4)	
C8	0.12183 (5)	0.7169 (3)	0.02175 (6)	0.0372 (4)	
C9	0.17098 (5)	0.6713 (3)	0.03861 (7)	0.0370 (4)	
C10	0.20563 (5)	0.4738 (3)	0.01377 (7)	0.0451 (4)	
H10A	0.1932	0.2840	0.0139	0.068*	
H10B	0.2352	0.4800	0.0356	0.068*	
H10C	0.2108	0.5304	−0.0242	0.068*	
C11	0.09468 (5)	0.5939 (3)	−0.02107 (6)	0.0368 (4)	
C12	0.04599 (5)	0.6554 (3)	−0.03528 (7)	0.0455 (4)	
H12	0.0304	0.7917	−0.0149	0.055*	
C13	0.02230 (6)	0.5199 (3)	−0.07759 (7)	0.0492 (4)	
H13	−0.0096	0.5601	−0.0856	0.059*	
C14	0.04580 (5)	0.3138 (3)	−0.11063 (7)	0.0435 (4)	
C15	0.02414 (6)	0.1660 (4)	−0.15629 (8)	0.0562 (5)	
H15	−0.0078	0.1959	−0.1664	0.067*	
C16	0.05019 (7)	−0.0228 (4)	−0.18596 (8)	0.0583 (5)	
H16	0.0357	−0.1184	−0.2164	0.070*	
C17	0.09766 (6)	−0.0735 (3)	−0.17137 (7)	0.0490 (4)	
H17	0.1146	−0.2024	−0.1921	0.059*	
C18	0.12003 (5)	0.0664 (3)	−0.12619 (7)	0.0404 (4)	
C19	0.09317 (5)	0.2600 (3)	−0.09639 (6)	0.0373 (4)	
C20	0.19362 (6)	−0.1685 (4)	−0.13639 (8)	0.0533 (5)	
H20A	0.1797	−0.3531	−0.1328	0.080*	
H20B	0.2255	−0.1704	−0.1201	0.080*	
H20C	0.1942	−0.1191	−0.1754	0.080*	

### Atomic displacement parameters (Å^2^)

**Table:**

	*U*^11^	*U*^22^	*U*^33^	*U*^12^	*U*^13^	*U*^23^
O1	0.0403 (7)	0.0647 (7)	0.0753 (9)	0.0165 (6)	0.0028 (6)	−0.0223 (7)
O2	0.0332 (6)	0.0421 (5)	0.0463 (7)	0.0051 (4)	0.0011 (5)	−0.0075 (5)
O3	0.0370 (6)	0.0583 (7)	0.0556 (7)	0.0076 (5)	0.0004 (5)	−0.0180 (6)
N1	0.0402 (7)	0.0393 (6)	0.0429 (8)	0.0073 (5)	0.0044 (6)	−0.0023 (6)
N2	0.0404 (8)	0.0400 (6)	0.0450 (8)	0.0098 (5)	0.0040 (6)	0.0006 (6)
C1	0.0522 (10)	0.0341 (7)	0.0372 (9)	0.0036 (6)	0.0069 (7)	0.0040 (7)
C2	0.0550 (11)	0.0476 (9)	0.0477 (10)	−0.0008 (7)	0.0044 (8)	−0.0021 (8)
C3	0.0697 (13)	0.0533 (10)	0.0496 (11)	−0.0105 (9)	0.0030 (9)	−0.0022 (9)
C4	0.0898 (16)	0.0451 (9)	0.0432 (11)	−0.0025 (9)	0.0052 (10)	−0.0037 (8)
C5	0.0816 (15)	0.0547 (10)	0.0501 (11)	0.0199 (9)	0.0104 (10)	−0.0039 (9)
C6	0.0592 (11)	0.0539 (9)	0.0491 (11)	0.0147 (8)	0.0022 (9)	−0.0044 (9)
C7	0.0380 (9)	0.0383 (7)	0.0472 (10)	0.0053 (6)	0.0059 (7)	0.0001 (7)
C8	0.0347 (8)	0.0346 (7)	0.0427 (9)	0.0040 (6)	0.0070 (7)	0.0010 (7)
C9	0.0369 (8)	0.0331 (7)	0.0413 (9)	0.0045 (6)	0.0063 (7)	0.0037 (7)
C10	0.0390 (9)	0.0433 (8)	0.0531 (10)	0.0080 (6)	0.0040 (7)	−0.0034 (7)
C11	0.0342 (8)	0.0340 (7)	0.0430 (9)	0.0046 (6)	0.0084 (7)	0.0028 (7)
C12	0.0368 (9)	0.0446 (8)	0.0554 (11)	0.0090 (7)	0.0066 (8)	0.0007 (8)
C13	0.0324 (9)	0.0537 (9)	0.0609 (11)	0.0080 (7)	−0.0020 (8)	0.0042 (8)
C14	0.0364 (9)	0.0443 (8)	0.0493 (10)	0.0028 (6)	−0.0020 (7)	0.0039 (7)
C15	0.0417 (10)	0.0619 (10)	0.0634 (12)	0.0029 (8)	−0.0131 (9)	0.0000 (9)
C16	0.0561 (11)	0.0632 (11)	0.0541 (12)	−0.0012 (9)	−0.0139 (9)	−0.0080 (9)
C17	0.0504 (10)	0.0507 (9)	0.0457 (10)	0.0016 (7)	0.0004 (8)	−0.0065 (8)
C18	0.0358 (9)	0.0419 (8)	0.0434 (9)	0.0002 (6)	0.0017 (7)	0.0006 (7)
C19	0.0366 (8)	0.0362 (7)	0.0388 (9)	−0.0014 (6)	0.0000 (7)	0.0013 (7)
C20	0.0453 (10)	0.0585 (10)	0.0565 (11)	0.0111 (8)	0.0060 (8)	−0.0096 (9)

### Geometric parameters (Å, º)

**Table:**

O1—C7	1.2299 (17)	C8—C9	1.438 (2)
O2—C11	1.3584 (17)	C9—C10	1.4931 (19)
O2—C19	1.3766 (17)	C10—H10A	0.9600
O3—C18	1.3578 (18)	C10—H10B	0.9600
O3—C20	1.4244 (18)	C10—H10C	0.9600
N1—C7	1.389 (2)	C11—C12	1.426 (2)
N1—N2	1.4046 (16)	C12—C13	1.341 (2)
N1—C1	1.419 (2)	C12—H12	0.9300
N2—C9	1.3048 (19)	C13—C14	1.433 (2)
C1—C6	1.382 (2)	C13—H13	0.9300
C1—C2	1.390 (2)	C14—C19	1.384 (2)
C2—C3	1.381 (2)	C14—C15	1.404 (2)
C2—H2	0.9300	C15—C16	1.372 (2)
C3—C4	1.376 (3)	C15—H15	0.9300
C3—H3	0.9300	C16—C17	1.385 (2)
C4—C5	1.367 (3)	C16—H16	0.9300
C4—H4	0.9300	C17—C18	1.385 (2)
C5—C6	1.391 (2)	C17—H17	0.9300
C5—H5	0.9300	C18—C19	1.401 (2)
C6—H6	0.9300	C20—H20A	0.9600
C7—C8	1.462 (2)	C20—H20B	0.9600
C8—C11	1.371 (2)	C20—H20C	0.9600
			
C11—O2—C19	121.38 (11)	C9—C10—H10C	109.5
C18—O3—C20	117.11 (12)	H10A—C10—H10C	109.5
C7—N1—N2	112.43 (12)	H10B—C10—H10C	109.5
C7—N1—C1	129.21 (13)	O2—C11—C8	116.14 (13)
N2—N1—C1	118.35 (12)	O2—C11—C12	118.14 (14)
C9—N2—N1	106.59 (12)	C8—C11—C12	125.72 (14)
C6—C1—C2	119.19 (15)	C13—C12—C11	121.15 (15)
C6—C1—N1	121.59 (15)	C13—C12—H12	119.4
C2—C1—N1	119.22 (14)	C11—C12—H12	119.4
C3—C2—C1	120.04 (16)	C12—C13—C14	120.59 (14)
C3—C2—H2	120.0	C12—C13—H13	119.7
C1—C2—H2	120.0	C14—C13—H13	119.7
C4—C3—C2	120.87 (18)	C19—C14—C15	118.31 (15)
C4—C3—H3	119.6	C19—C14—C13	117.17 (14)
C2—C3—H3	119.6	C15—C14—C13	124.52 (15)
C5—C4—C3	118.99 (17)	C16—C15—C14	119.84 (16)
C5—C4—H4	120.5	C16—C15—H15	120.1
C3—C4—H4	120.5	C14—C15—H15	120.1
C4—C5—C6	121.25 (18)	C15—C16—C17	121.19 (16)
C4—C5—H5	119.4	C15—C16—H16	119.4
C6—C5—H5	119.4	C17—C16—H16	119.4
C1—C6—C5	119.64 (18)	C16—C17—C18	120.50 (16)
C1—C6—H6	120.2	C16—C17—H17	119.7
C5—C6—H6	120.2	C18—C17—H17	119.7
O1—C7—N1	125.57 (15)	O3—C18—C17	125.90 (14)
O1—C7—C8	130.78 (15)	O3—C18—C19	116.25 (13)
N1—C7—C8	103.65 (12)	C17—C18—C19	117.84 (14)
C11—C8—C9	129.57 (13)	O2—C19—C14	121.56 (13)
C11—C8—C7	124.98 (13)	O2—C19—C18	116.12 (13)
C9—C8—C7	105.45 (13)	C14—C19—C18	122.31 (14)
N2—C9—C8	111.87 (13)	O3—C20—H20A	109.5
N2—C9—C10	119.63 (13)	O3—C20—H20B	109.5
C8—C9—C10	128.50 (14)	H20A—C20—H20B	109.5
C9—C10—H10A	109.5	O3—C20—H20C	109.5
C9—C10—H10B	109.5	H20A—C20—H20C	109.5
H10A—C10—H10B	109.5	H20B—C20—H20C	109.5
			
C7—N1—N2—C9	−0.36 (16)	C19—O2—C11—C12	−0.2 (2)
C1—N1—N2—C9	−179.67 (12)	C9—C8—C11—O2	0.7 (2)
C7—N1—C1—C6	5.1 (2)	C7—C8—C11—O2	−179.45 (13)
N2—N1—C1—C6	−175.68 (14)	C9—C8—C11—C12	−179.11 (15)
C7—N1—C1—C2	−174.84 (15)	C7—C8—C11—C12	0.8 (2)
N2—N1—C1—C2	4.3 (2)	O2—C11—C12—C13	1.2 (2)
C6—C1—C2—C3	−0.8 (2)	C8—C11—C12—C13	−179.03 (15)
N1—C1—C2—C3	179.12 (14)	C11—C12—C13—C14	−1.5 (2)
C1—C2—C3—C4	−0.4 (3)	C12—C13—C14—C19	0.8 (2)
C2—C3—C4—C5	1.1 (3)	C12—C13—C14—C15	−178.71 (16)
C3—C4—C5—C6	−0.6 (3)	C19—C14—C15—C16	−1.0 (3)
C2—C1—C6—C5	1.4 (2)	C13—C14—C15—C16	178.53 (16)
N1—C1—C6—C5	−178.61 (14)	C14—C15—C16—C17	0.7 (3)
C4—C5—C6—C1	−0.7 (3)	C15—C16—C17—C18	0.0 (3)
N2—N1—C7—O1	−179.78 (15)	C20—O3—C18—C17	−2.8 (2)
C1—N1—C7—O1	−0.6 (3)	C20—O3—C18—C19	177.68 (14)
N2—N1—C7—C8	0.35 (16)	C16—C17—C18—O3	−179.84 (15)
C1—N1—C7—C8	179.58 (13)	C16—C17—C18—C19	−0.3 (2)
O1—C7—C8—C11	0.0 (3)	C11—O2—C19—C14	−0.5 (2)
N1—C7—C8—C11	179.86 (14)	C11—O2—C19—C18	178.69 (12)
O1—C7—C8—C9	179.92 (17)	C15—C14—C19—O2	179.76 (14)
N1—C7—C8—C9	−0.22 (15)	C13—C14—C19—O2	0.2 (2)
N1—N2—C9—C8	0.20 (16)	C15—C14—C19—C18	0.6 (2)
N1—N2—C9—C10	−179.44 (12)	C13—C14—C19—C18	−178.94 (14)
C11—C8—C9—N2	179.93 (14)	O3—C18—C19—O2	0.4 (2)
C7—C8—C9—N2	0.02 (17)	C17—C18—C19—O2	−179.14 (13)
C11—C8—C9—C10	−0.5 (3)	O3—C18—C19—C14	179.59 (14)
C7—C8—C9—C10	179.61 (14)	C17—C18—C19—C14	0.0 (2)
C19—O2—C11—C8	−179.97 (12)		

### Hydrogen-bond geometry (Å, º)

Cg1 and Cg2 are the centroids of the N1/N2/C7–C9 and C11–C14/C19/O2 rings, respectively.

**Table:**

*D*—H···*A*	*D*—H	H···*A*	*D*···*A*	*D*—H···*A*
C6—H6···O1	0.93	2.28	2.911 (2)	124
C12—H12···O1	0.93	2.38	3.004 (2)	124
C13—H13···O1^i^	0.93	2.53	3.2577 (19)	136
C10—H10*A*···*Cg*1^ii^	0.96	2.79	3.6812 (17)	155
C7—O1···*Cg*2^iii^	1.23 (1)	3.65 (1)	3.9797 (18)	96 (1)

Symmetry codes: (i) −*x*, −*y*+2, −*z*; (ii) *x*, *y*−1, *z*; (iii) *x*, *y*+1, *z*.
