# Supplementary figures and images for: Crystal structure of (4E)-4-(8-meth­oxy-2H-chromen-2-yl­idene)-3-methyl-1-phenyl-1H-pyrazol-5(4H)-one
Source: Acta Crystallogr E Crystallogr Commun. 2015 May 23;71(Pt 6):o414–5. doi: 10.1107/S2056989015009445 (PMC4459315; doi:10.1107/S2056989015009445)

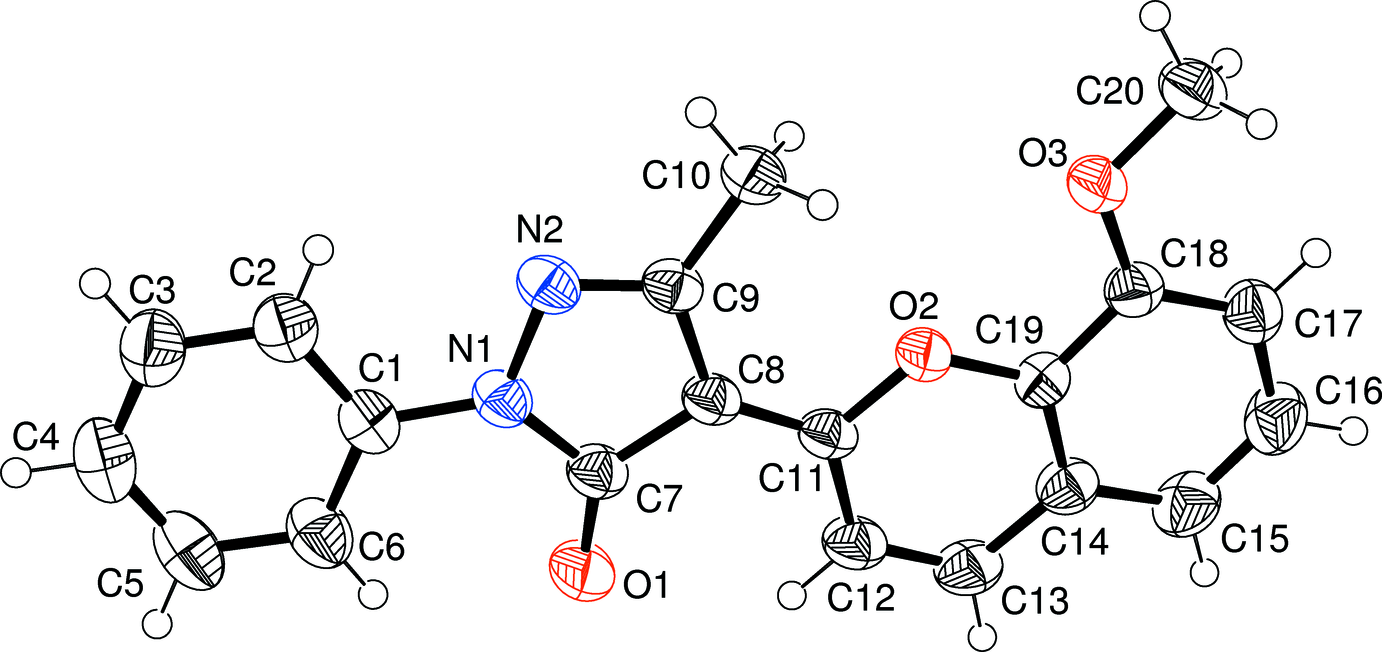

Supplement: Supplementary file 4 [file e-71-0o414-fig1.tif]

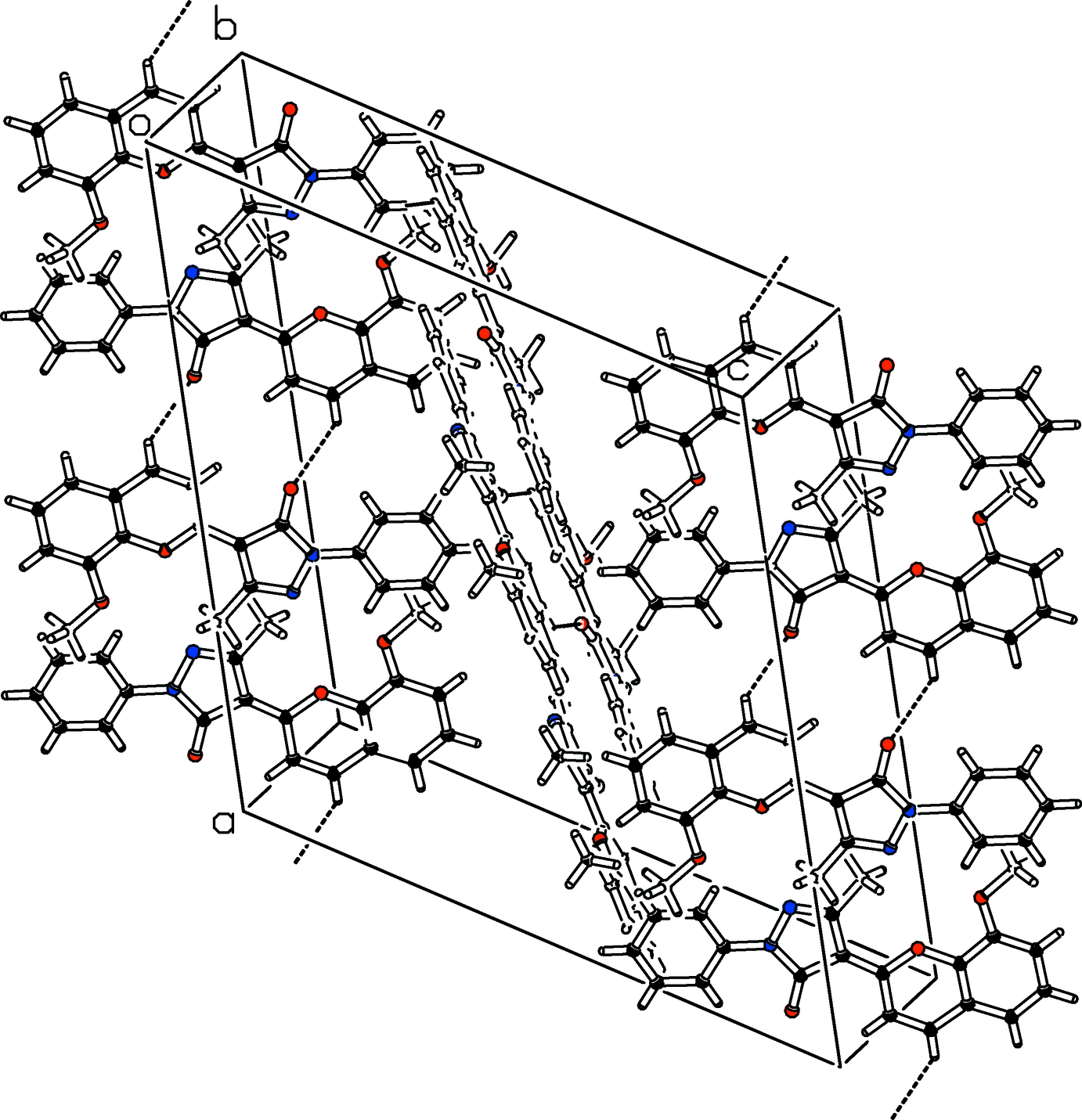

Supplement: Supplementary file 5 [file e-71-0o414-fig2.tif]
